# Look away: arterial and venous intravascular embolisation following shotgun injury

**DOI:** 10.1186/1752-2897-8-19

**Published:** 2014-11-15

**Authors:** John Vedelago, Elizabeth Dick, Robert Thomas, Brynmor Jones, Olga Kirmi, Jennifer Becker, Afshin Alavi, Wladyslaw Gedroyc

**Affiliations:** Radiology Department, St. Mary’s Hospital, Praed Street, London, W2 1NY United Kingdom

**Keywords:** Firearm trauma, Ballistic trauma, Trauma management, Vascular injuries, Pellet embolisation, Computed tomography, Shotgun

## Abstract

We describe two cases of intravascular embolization of shotgun pellets found distant to the entry site of penetrating firearm injury. The cases demonstrate antegrade embolization of a shotgun pellet from neck to right middle cerebral artery, and antegrade followed by retrograde venous embolization through the left lower limb to pelvis. Radiologists and Trauma Physicians should be aware that post shotgun injury, the likelihood of an embolised shot pellet is increased compared to other types of firearm missile injury, and should therefore search away from the site of injury to find such missiles. Shotgun pellets may travel in an antegrade or a retrograde intravascular direction – both were seen in these cases - and may not be clinically obvious. This underscores the importance of a meticuluous search through all images, including CT scout images, for evidence of their presence.

## Findings

Intravascular embolisation of a bullet or metallic pellet is a rare but potentially catastrophic complication of shotgun injury. It is important that Trauma Physicians and Radiologists are aware of the complications that may arise from this type of injury and its increased frequency in shotgun injury relative to other types of gun-related trauma. We present two cases from a Level 1 (Major) Trauma Centre in the United Kingdom seen within a two-month period. In Case 1, arterial embolisation of a pellet to the right middle cerebral artery (MCA) resulted in massive MCA territory infarction whilst in Case 2, antegrade followed by retrograde venous embolisation of a shot fragment demonstrated pellet migration from the lower thigh to pelvis.

### Case 1

**Figure 1:**
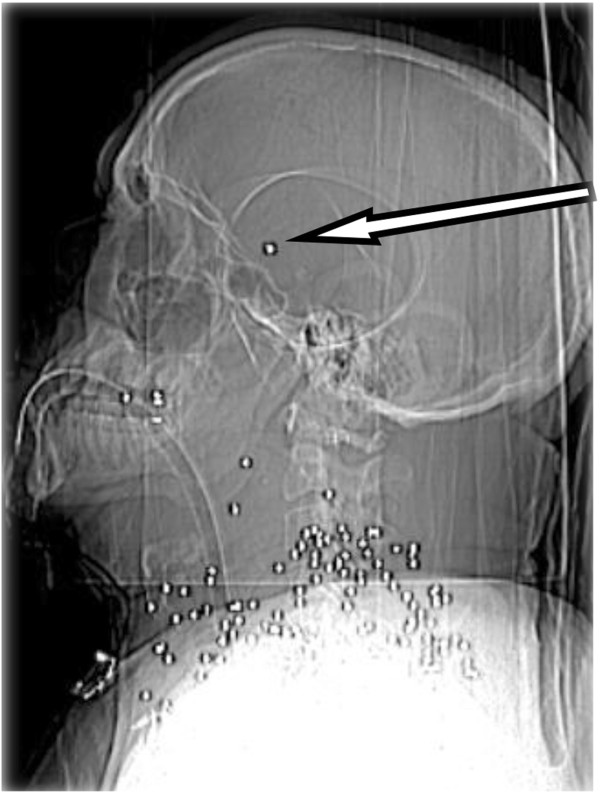
**Lateral scout CT head and neck image at presentation.**

**Figure 2:**
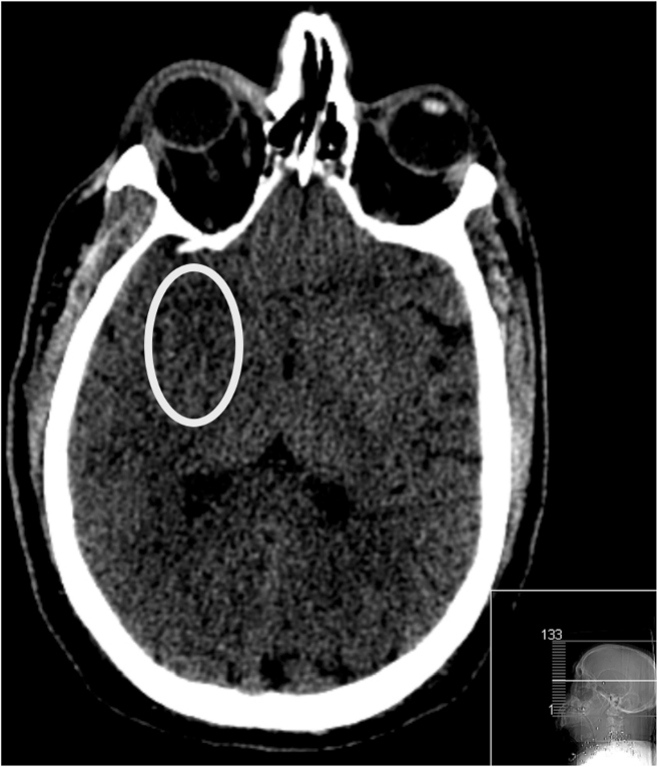
**Axial image from non-contrast CT brain at presentation.**

**Figure 3:**
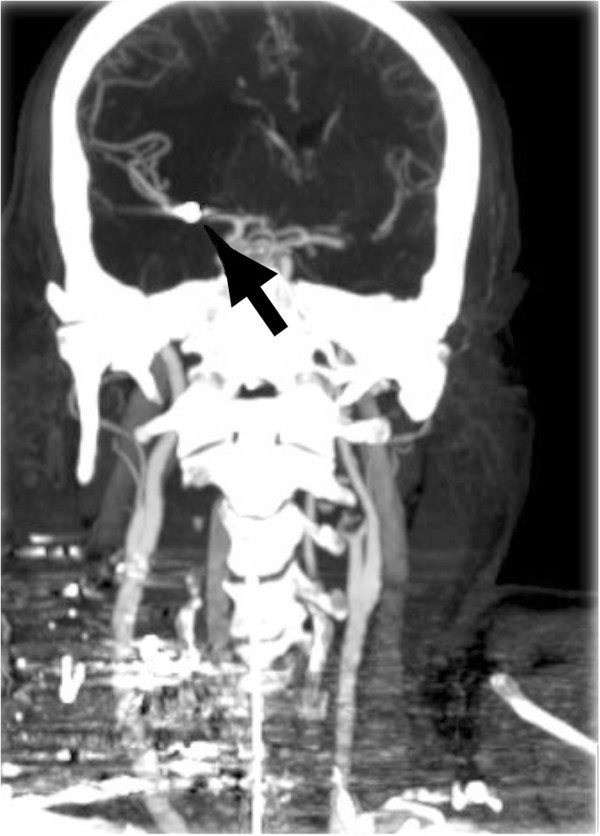
**CT angiography at 24 hours post presentation.**

A 55 year old male with low GCS was intubated and transferred to our level 1 trauma centre following a single shotgun bullet wound to the right anterior neck and supraclavicular region. He had been documented to be hypotensive post injury by ambulance first responders. CT was performed approximately three hours after the injury and demonstrated scores of spherical metallic shot pellets scattered over a 15 cm × 15 cm area of the neck and supraclavicular region. The CT head lateral scout revealed a single intracranial pellet, projected over the pterion (Figure 1 arrow). Non-contrast CT demonstrated early signs of an acute MCA infarct with loss of the insula ribbon grey-white matter differentiation and subtle hypoattenuation within the putamen and globus pallidus (Figure 2 white oval). Subsequent CT angiography of intracranial vessels confirmed an intra-arterial 3 mm pellet within the M1 segment of the right middle cerebral artery resulting in moderate flow reduction. Follow-up CT angiography performed 24 hours after arrival showed complete reperfusion of the right middle cerebral artery (Figure 3) with the shot fragment seemingly unchanged in position (arrow).

**Figure 4:**
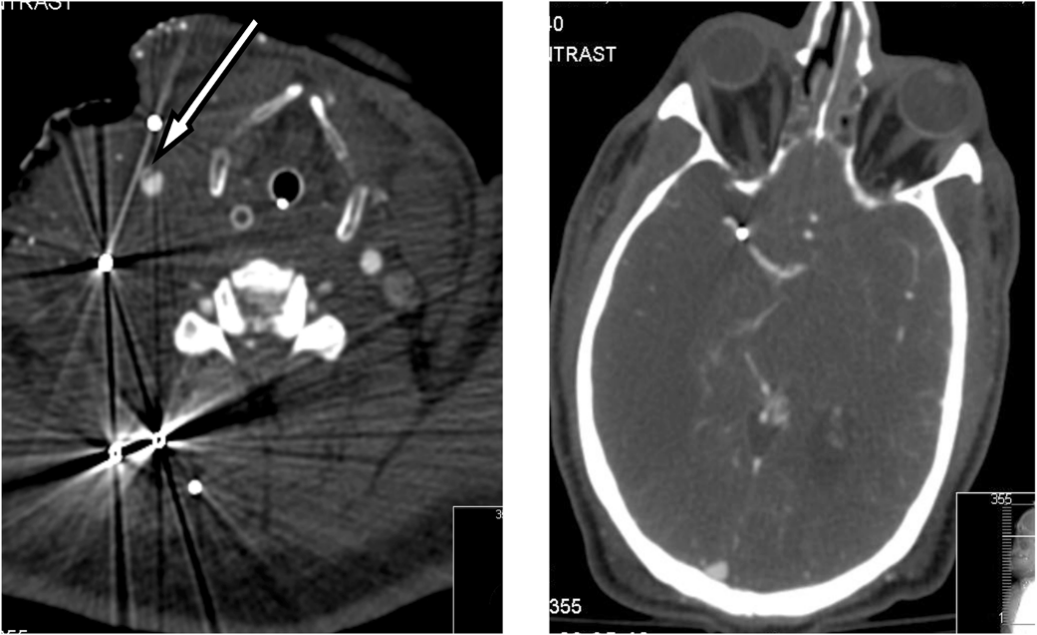
**Axial images CT carotid and Circle of Willis angiogram.**

CT angiography of the neck revealed a focal dissection (Figure 4 arrow) and linear focus of thrombus within the right common carotid artery 5 cm inferior to the bifurcation, at the presumed arterial entry point of the embolised pellet. A large surrounding haematoma, but no active arterial extravasation of contrast, was present. The pulmonary venous system, a second potential entry site described [1], was unremarkable.

The patient developed a large right-sided MCA territory infarct involving the entirety of the right middle cerebral artery territory. Craniectomy was performed to reduce intracranial hypertension and mass effect due to profound parenchymal swelling. An enterostomy tube was required for feeding and the patient remained an inpatient several months after admission.

### Case 2

**Figure 5:**
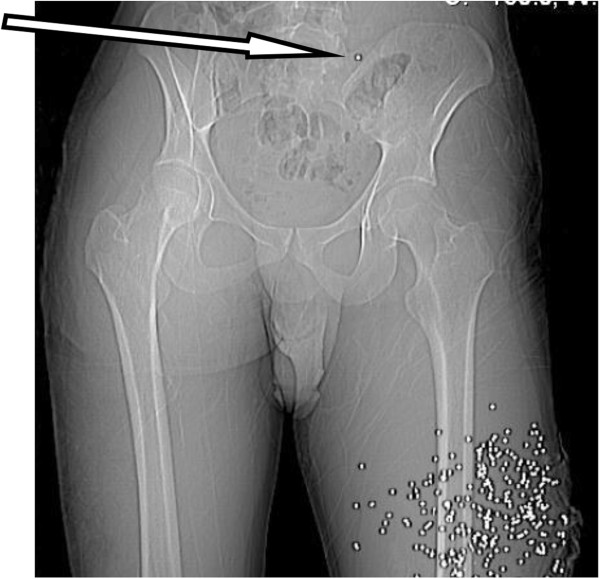
**CT scout view pelvis and upper thigh.**

**Figure 6:**
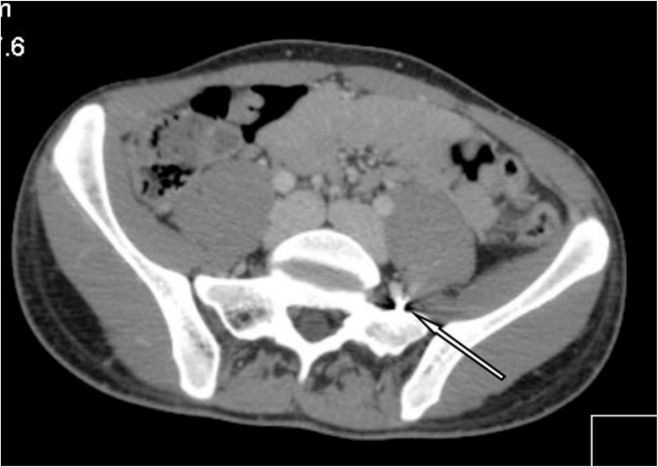
**Axial post contrast CT image.**

A 21 year old male was transferred to our level 1 trauma centre after sustaining a shotgun wound to the lower left thigh. Radiographs of the pelvis and CT angiography were obtained at presentation. A 2 mm rounded metallic shot fragment was seen to lie within the proximal right iliolumbar vein, with the pellet projected over the sacrum on the AP supine radiograph and CT scout images (Figure 5 arrow). The pellet was seen to lie within the left iliolumbar vein (Figure 6 arrow), distant to the site of penetrating firearm trauma. There was no clinical or radiological evidence of thoracic, abdominal or pelvic wall trauma, and there were no pellets within the pulmonary arterial tree visible on chest radiograph. The combination of findings were consistent with antegrade embolisation of a shot pellet from the femoral to the external then common iliac veins, with subsequent retrograde passage in to the left iliolumbar vein, due to the effect of gravity and patient’s supine position following the injury.

## Discussion of findings

Shotgun pellets are the most likely of the firearm missiles to result in intravascular embolisation. Da Costa et al. [2] state several reasons for this: metallic ‘shot’ pellets usually cover a wide area with a relatively large number of pellets penetrating the soft tissues and that for embolisation to occur, the diameter of the injured vessel must be greater than that of the pellet. In bullet trauma, this is often not the case. A further predisposing factor is that the velocity and energy dissipation of the pellets must be high enough to puncture one rather than both sides of the vessel wall, a phenomenon bullets are less likely to exhibit.

Intravascular embolization of shotgun pellets or single bullets has been previously described to the brain, with or without catastrophic sequelae [3–8]. Whilst retrograde intravascular embolisation of intravascular missiles is a relatively infrequently described event [9–12], the radiologist and emergency department physician involved in assessment of firearm injury must be aware of the increased probability of both antegrade and retrograde intravascular embolisation in shotgun trauma. Missile embolization may not be suspected at the time of injury [13] or may be delayed [14]. Shotgun injury should therefore trigger an automatic and meticulous search of all cross sectional imaging for the presence of embolised pellets or fragments. CT scout images may be particularly helpful in revealing pellets in regions not imaged on initial x-ray series. A search for direct evidence of vessel wall injury is also indicated, and yielded the site of vascular penetration in case 1.

Case 1 illustrates that, depending on the timing of imaging post MCA-shot pellet embolisation occurs, flow within the vessel may be reduced or increased in the presence of a shot pellet. There are a number of postulated potential mechanisms which may contribute to the infarction demonstrated in this case, despite the presence of increased flow on the CT angiography described. There may have been temporary vessel occlusion caused by the pellet itself, at a site of anatomical vessel diameter narrowing or partial stenosis, followed by relative hyperaemia due to altered autoregulation post ischaemia/infarction present at the time of the CTA. The lodged bullet may also have induced temporary vasospasm, reducing or preventing arterial flow, which had improved by the time of CTA. Transient hypotension, as occurred in patient 1 prior to resuscitation is also a potential factor in exacerbating reduced perfusion. Finally, the presence of an intra-arterial fragment may act as a potential nidus for thrombus formation, although there was no evidence of such in this particular case.

## Consent

Written informed consent was obtained from the author for the publication of this report and any accompanying images.
